# Understanding the computational insights of spin-polarised density functional theory into the newly half-metallic *f* electron-based actinide perovskites SrMO_3_ (M = Pa, Np, Cm, Bk)

**DOI:** 10.1038/s41598-023-43624-7

**Published:** 2023-10-06

**Authors:** Sakshi Gautam, Sukriti Ghosh, Dinesh C. Gupta

**Affiliations:** Condensed Matter Theory Group, School of Studies in Physics, Jiwaji University Gwalior, Gwalior, MP 474001 India

**Keywords:** Materials science, Physics

## Abstract

Here, we investigated the structural, mechanical, electronic, magnetic, thermodynamic and thermoelectric properties of Strontium based simple perovskites SrMO_3_ (M = Pa, Np, Cm, Bk) by using density functional theory. First and foremost, the ground state stability of these perovskites was initially evaluated by optimizing their total ground state energies in distinct ferromagnetic and non-magnetic configurations. The structural stability in terms of their ground state energies defines that these alloys stabilize in ferromagnetic rather than competing non-magnetic phase. From the understandings of mechanical parameters these alloys are characterized to be ductile in nature. After that, two approximation schemes namely Generalized Gradient approximation and Tran-Blaha modified Becke-Johnson potential have been used to find their intimate electronic structures which displays the half-metallic nature of these alloys. Further, we have verified temperature and pressure effect on these alloys. Finally, the transport properties have been evaluated within the selected temperature range of 150–900 K. In view of this, the different transport parameters along with half-metallic nature advocate their possible applications in thermoelectric and spintronics devices.

## Introduction

Nowadays, the materials having half- metallic nature are at top billing, because of their overwhelming properties. These materials are highly applicable in different domains like magnetic tunnel junction, spintronics^1^, thermoelectrics, etc. Half-metallic materials are the materials which show metallic behavior in one channel while semiconducting or insulating in second channel. These materials show 100% spin-polarization at the Fermi-level^2^. Due to this feature they are responsible to generate highly spin polarized current. The antiparallel magnetization configuration of half-metallic compounds layers therefore would exhibit infinitely high resistance, whereas the parallel configuration demonstrates zero resistance. Hence, these materials are thus expected to produce extremely high magnetoresistance required for GMR devices. Thereby, would significantly improve the efficiency of spintronic devices. Recently, different alloys like Perovskites^3^, double-perovskites^4^, Heusler-alloys, transition metal oxides, chalcogenides, etc. have been reported to show half-metallic nature. Among them perovskites materials are the center piece for the researchers because of their various properties like colossal magnetoresistance^5^, piezoelectricity^6^, ferroelectricity^7^, charge-ordering, superconductivity, multiferroicity, etc. Designing new perovskites as well as tailoring available perovskites to obtain optimized properties is still an active research area. In nature perovskites are mainly found as oxides having general formula ABX_3_ where A and B are cations and X is an anion usually oxides or halogens whereas A is alkali or alkaline element and B is either transition or inner transition element, sometimes from s block also^8,9^. Currently, extensive research is perceived rigorously either theoretically or experimentally to see these functional alloys for many perspective needs. The present work has been established on the perovskite systems SrMO_3_ (M = Pa, Np, Cm, Bk) to examine the various properties and applicability in various spheres. In the recent past, many lanthanides and actinide-based ABO_3_ perovskites have been studied and most of them display the half-metallic nature because of the localized *f*-states. This results in high spin-polarization at the Fermi-level hence, gives a way to spintronics field. Shakeel et al. have worked on BaNpO_3_ perovskite^10^, M. Nabi et al. have reported about BaBkO_3_ alloy^11^ and they concluded that these materials possess half-metallic nature and is quite applicable for thermoelectric and spintronic perspectives. Also, Zahid et al. explored the physical properties of BaXO_3_ (X = Pr, U) compounds with the help of DFT and predicted that the half-metallic nature is preserved within these perovskites^12^. Besides this, Sajad et al. reported SrAmO_3_, compound as thermodynamically stable and of half-metallic^13^. In addition, alloys based on inner transition like CsEuCl_3_, CsYbCl_3_, RbYbF_3_ CsTmCl_3_, etc_._ were investigated to check their applicability in optoelectronics realm also and their study reveals that this class of alloys are suitable for the field of optoelectronics and optical devices as well^14–18^. Further, the researchers from all over the world are searching new alternative energy resources as it is a major concern to be resolved because the natural non-renewable energy resources are limited and have created environmental issues like global warming, degrading the quality of air etc. Inner transition-based perovskites showcase its applications in thermoelectric realm also by converting waste heat into useful energy through Seebeck effect^19–23^. Moreover, actinide-based perovskites have grabbed attention for their applications in radioisotope generator (RTG’s). So, on the behalf of the mentioned literature, we have carried the same DFT calculations first time to investigate the physical properties of Strontium-based oxides viz. SrPaO_3_, SrNpO_3_ and SrCmO_3_ and SrBkO_3_ by using FP-LAPW + lo scheme.

## Computational details

To estimate the various physical properties of these alloys, first principle DFT calculations were suitably executed by adopting the Full potential linearized augmented plane wave (FP-LAPW)^24^. However, the process of DFT calculations begins with simple GGA^25^ to testify the intimate electronic band structure as well as density of states. As it is well known that GGA underestimates the electronic structure especially if the system involves d/f electrons. So, staying within the DFT, we have implemented GGA + mBJ scheme to verify the results^26^. The muffin tin radii of Sr, Pa, Np, Cm and O atoms are 0.132, 0.13, 0.11, 0.11 and 0.09 (nm) respectively. Potential and charge density non- spherical contributions to muffin tin (MT) spheres were expanded to l_max_ = 10 and convergence criteria for energy and charge were set to 10^–4^ Ry and 10^–4^ eV respectively. An ample mesh of 3000 k points is used for Brillouin zone integration through modified tetrahedron method. The package that we used here for the determination of the mechanical strength of these alloys, for various structures is cubic elastic package which is energy approach interface embedded in Wien2k simulation code^27^. The pressure and temperature effect on various thermodynamic quantities has been explored from Gibbs 2 code to figure out the stability^28^. Whereas, to evaluate the different transport coefficients, semiclassical Boltzmann theory within BoltzTrap scheme^29^ has been utilized. The k-point mesh is raised to 150, 000 for better convergence and to obtain accurate transport coefficients.

## Discussion on results

The results of different properties for these materials are summarized below;

### Structural properties

**Figure 1:**
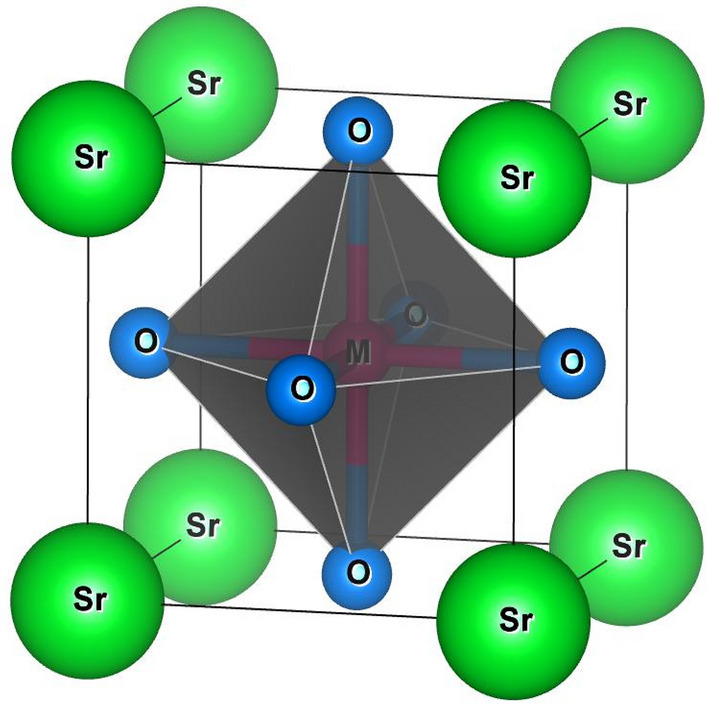
Molecular crystal structure of SrMO_3_(M = Pa, Np, Cm, Bk) alloys.

**Figure 2:**
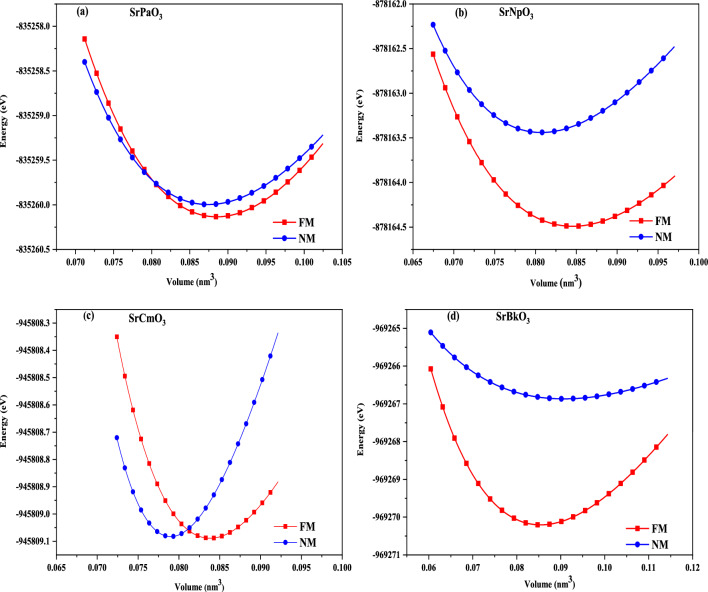
(**a-d**) Optimization plots of SrMO_3_ (M = Pa, Np, Cm, Bk) alloys in ferromagnetic and non-magnetic phases.

The structural properties of a material provide us keen understanding regarding the location of atoms at distinct positions. Here, the unit cell structure of these perovskites has been shown in Fig. 1. These alloys crystallize in the Pm-3 m (221) space group with Sr, M and O atoms positioned at corners, body centre and face centre respectively while, the Wyckoff positions of individual atoms are descripted as Sr resides at (0, 0, 0) while (0.5,0.5,0.5) for Pa, Np, Cm, Bk and (0.5,0.5,0) for O atom. This defines the complete geometry of these atoms, where the atoms are residing at respective positions. Next, to extract the ground state structural parameters these alloys were simulated in FM and NM magnetic configurations by employing the art of Birch-Murnaghan’s equation of state^30^. The optimized energy-volume curve in these different phases clearly indicates that FM phase is significantly most stable as it holds lowest energy rather than competing NM phase as depicted in Fig. 2. Subsequently, several physical parameters inclusive of lattice parameters (a_0_), bulk modulus (B), the derivative of bulk modulus (B^´^), and Volume (V) of these alloys have been retrieved as displayed in Table 1. In the obtained parameters, the bulk modulus (B) and pressure derivative of the bulk modulus (B^’^) plays a very important role while designing materials. Mathematically, expressed as $B=-V(\frac{\partial P}{\partial V})$_T_ and $B^{\prime}=-V(\frac{\partial B}{\partial V})$_T._ Here, the bulk modulus depicts the material’s capability to withstand volume changes when pressure is applied from all sides. Whereas, the pressure derivative of the bulk modulus gives an idea of the rate at which bulk modulus expands on increasing pressure. It is a dimensionless parameter, important for determining thermoelastic properties of materials at higher temperatures and pressures^31^. As no experimental data is found for these alloys so to accord the data of obtained parameters, we have compared the results with similar alloys like KNpO_3_, KPuO_3_, RbNpO_3_ and RbPuO_3_^32,33^ which are in fine agreement with the previously published results as illustrated in Table 2. In addition to that, the structural stability has been further evaluated to see the most probable ground state structure for which we have unanimously used the Goldsmith’s tolerance factor “*t”* relation which is commonly enumerated as;

$$t = 0.{7}0{7}\left( {\frac{{r_{{A + r_{O} }} }}{{r_{B} + r_{O} }}} \right)$$

where, ${r}_{A}$, ${r}_{B}$ and be the radius of cations and ${r}_{O}$ is the radius of anion. For *t* in the range of 0.93–1.04^34^ alloy possess the cubic structure. The obtained values of the tolerance factor for these alloys are listed in Table 1 concludes that its value falls in the given range hence, affirmed the stability in cubic phase for these alloys.

**Table 1:** Calculated lattice parameters (a_o_), minimum free energy (E_0_), bulk modulus (B), the derivative of bulk modulus (B’^´^), Volume (V) and cohesive energy (eV/atom) for SrMO_3_ alloys (M: Pa, Np, Cm, Bk).

Parameters	SrPaO_3_	SrNpO_3_	SrCmO_3_	SrBkO_3_
a_o_ (nm)	0.45	0.44	0.43	0.44
E_0_ (eV)[FM]	−835,260.04	−878,164.37	−945,809.00	−969,270.20
E_0_ (eV)[NM]	−835,259.91	−878,163.42	−945,805.47	−969,266.83
B (GPa)	144.15	123.10	103.40	117.18
B’	2.82	4.07	6.9	3.0
Volume (nm^3^)	0.080	0.079	0.078	0.085
T	0.9	0.9	0.9	0.9
E_Coh_	5.68	5.00	4.08	3.57

**Table 2:** Calculated results of other perovskites for comparison.

Parameters	KNpO_3_	KPuO_3_	RbNpO_3_	RbPuO_3_
E_0_ (eV)[FM]	−808,049.44	−830,150.58	−872,767.84	−894,867.97
B (GPa)	131.00	122.76	129.80	126.40
B’	4.37	4.67	4.55	4.73
C_11_	279.12	261.62	260.57	235.15
C_12_	56.42	53.57	69.87	68.71
C_44_	50.73	47.77	42.50	40.64

Further, to substantiate the stability of these alloys we have computed the cohesive energy which is the energy required to separate the components from the crystal. Mathematically, it can be represented as: ${E}_{c}=\frac{\left[x{E}_{Sr}+y{E}_{M}+z{E}_{O}\right]-{E}_{Total}}{x+y+z}$. Here, E_Total_ represents total optimized energy while E_Sr_, E_M_ and E_O_ are the energies of isolated Sr, M (Pa, Np, Cm, Bk) and O atoms, respectively and x, y and z are numbers of Sr, M and O constituents in a conventional unit cell, respectively. The recorded values are listed in same Table 1 which are positive for all the four materials. Hence, indicate that these alloys can be synthesized experimentally^4^.

### Mechanical properties

In this study we have worked with cubic system it requires only three ${C}_{11}$*,* ${C}_{12}$ and ${C}_{44}$ elastic constants. For a cubic structure, the necessary conditions to find the mechanical stability are (C_11_ − C_12_) > 0, C_11_ > 0, C_44_ > 0, (C_11_ + 2C_12_) > 0, C_12_ < B < C_11_^35^, the titled alloys follow the mentioned conditions thus endorse the stability of these alloys. The values of C_ij_ constants are listed in Table 3. By using the values of these elastic constants, we have calculated various other elasto-mechanical parameters like Young’s, Bulk and shear moduli, etc. which are also enlisted in Table 3. Viogt-Reuss-Hill method^36^ is used to obtain the bulk and shear moduli. The Viogt constraints of the B_V_ and G_V_ are expressed as ${B}_{V}=(\frac{{C}_{11}+{2C}_{12}}{3}$) and ${G}_{V=}(\frac{{C}_{11}-{C}_{12}+{3C}_{44}}{5}$).

**Table 3:** Calculated elasto-mechanical parameters at 0 GPa and 0 K.

Alloy	C11	C12	C44	G	B	Y	Ν	A	B/G	C’’
SrPaO_3_	238.94	69.63	31.92	47.76	126.06	127.22	0.33	0.37	2.63	37.71
SrNpO_3_	230.22	70.16	34.27	48.5	123.51	128.66	0.32	0.42	2.54	35.89
SrCmO_3_	219.33	64.88	45.3	56.17	116.36	145.15	0.29	0.58	2.07	19.58
SrBkO_3_	235.91	56.54	39.47	55.21	116.33	143.01	0.29	0.44	2.10	17.07

While, the bulk modulus and shear modulus by Reuss formulism are expressed as:

$$\mathrm{B_{V} }=\mathrm{ B_{R}\; and }\;{G}_{R}=\frac{5({C}_{11}-{C}_{12)}{C}_{44}}{{4C}_{44}+3{(C}_{11-}{C}_{12})}$$

whereas, the bulk and shear moduli by Hill’s approximation can be expressed as:

$$B=\frac{{(B}_{V}+{B}_{R})}{2}\; \mathrm{and }\;G=\frac{{(G}_{V}+{G}_{R})}{2}$$

Here, we have also calculated Young Modulus by using the values of B and G enumerated as:

$$Y = \frac{9BG}{{3B + G}}$$

It represents the ratio of normal stress to longitudinal strain and quantifies an elastic solid's resistance to a change in length. Further, we have also calculated the Poisson’s ratio for these alloys which helps to understand the nature of bonding forces, the obtained values are enlisted in the same Table 3. Straight from the elastic constants one can deduce anisotropic factor $$A=\frac{{2C}_{44}}{{C}_{11}-{C}_{12}}$$ which is helpful in charactering anisotropic nature of material and for these alloys its value is greater than 1, hence, indicating that they are anisotropic in nature^37^, therefore elastic waves have different velocities in different directions. By using Bugger’s relation^38^ we have evaluated the magnitude of longitudinal ($${v}_{l}$$) and transverse waves ($${v}_{t1 }\;and\; {v}_{t2})$$ along (100), (110), (111) directions enlisted in Table 4. For these alloys we have also calculated the Pugh’s ratio (B/G) to ensure the nature of material whether the material is ductile or brittle which comes out to be greater than 1.75^39^ so indicating the ductile nature of the alloys. Further, another parameter Cauchy’s relation defined as C’’ = (C_12_–C_44_) also clarifies the nature of the material. In this study because we get its positive value hence, supports the ductile nature of these alloys^11^. Moreover, for the titled alloys we have calculated Debye temperature by using the equation given as $${\theta }_{D}=\frac{h}{k}{\left(\frac{3n\rho {N}_{A}}{4\pi m}\right)}^\frac{1}{3}{v}_{m}$$ where h is Plank’s constant, k is Boltzmann’s constant, N_A_ is Avogadro number, ρ is density and $${v}_{m}$$ is average sound velocity which can be calculated as:$${v}_{m}{=[\frac{1}{3}\left(\frac{2}{{v}_{l}3} +\frac{1}{{v}_{t}3}\right)]}^{\frac{-1}{3}}$$. To find $${v}_{m },$$ we have estimated longitudinal velocity ($${v}_{l })$$ and transverse velocity ($${v}_{t})$$ by using Navier’s equation^40^ as given $${v}_{l}$$=$$\sqrt{\frac{3B+4G}{\rho }}$$ and $${v}_{t}$$= $$\sqrt{\frac{G}{\rho }}$$. The obtained values of longitudinal velocity, transverse velocity, mean velocity and Debye temperature θ_D_ are listed in Table 5.

**Table 4:** Calculated sound (m/s) and averaged velocities along different directions.

Alloy	$\nu_{l}$			$\nu_{{t_{1} }}$			$\nu_{{t_{2} }}$			$\nu_{m}$		
Planes	[100]	[110]	[111]	[100]	[110]	[111]	[100]	[110]	[111]	[100]	[110]	[111]
SrPaO_3_	2948	2603	2477	1078	1755	1562	1078	1755	1562	1215	1413	1707
SrNpO_3_	2805	2511	2405	1082	1654	1489	1082	1654	1489	1219	1399	1630
SrCmO_3_	2690	2487	2415	1223	1596	1482	1223	1596	1482	1369	1507	1625
SrBkO_3_	2817	2499	2384	1152	1152	1566	1152	1152	1566	1295	1479	1704

**Table 5:** The calculated longitudinal, transverse, mean velocities (m/s) and Debye Temperature (K).

Alloy	*v*_t_	*v*_*l*_	*v*_*m*_	θ_D_
SrPaO_3_	1318	2627	1468	223
SrNpO_3_	1288	2536	1434	220
SrCmO_3_	1361	2512	1509	234
SrBkO_3_	1362	2527	1511	232

### Electronic and magnetic properties

**Figure 3:**
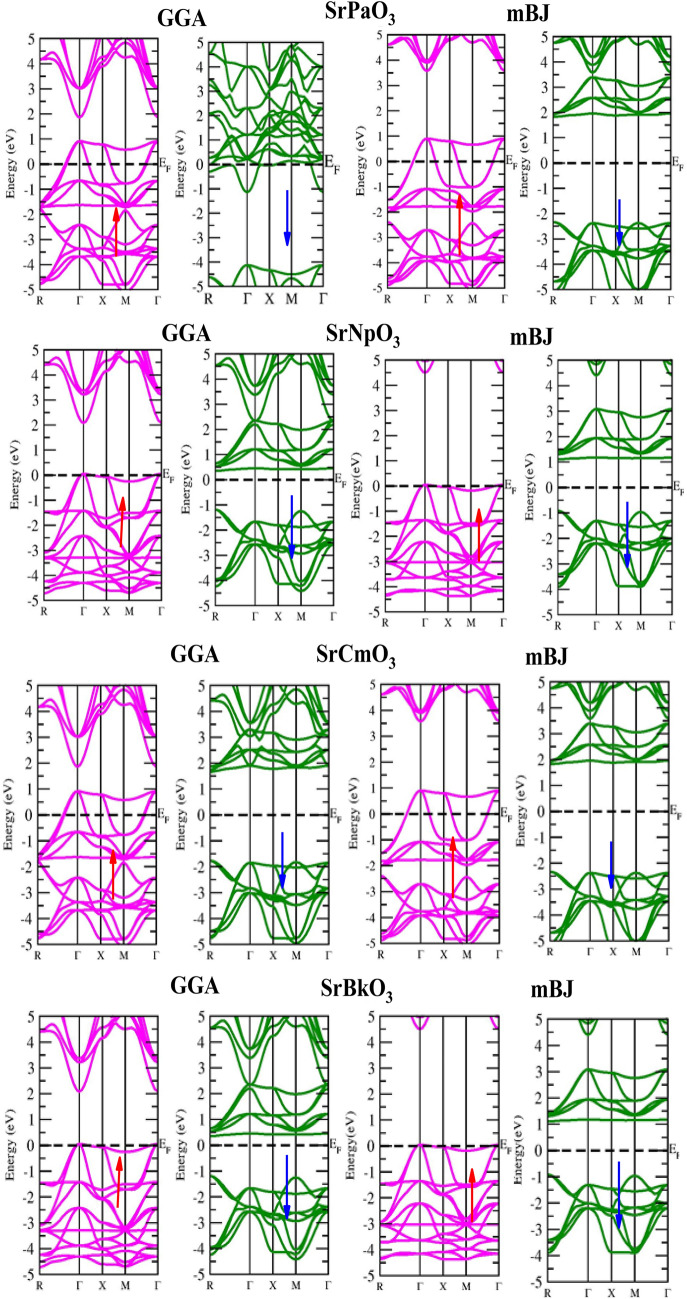
Band structure of SrMO_3_ (M = Pa, Np, Cm, Bk) alloys by GGA GGA + mBJ approximation.

**Figure 4:**
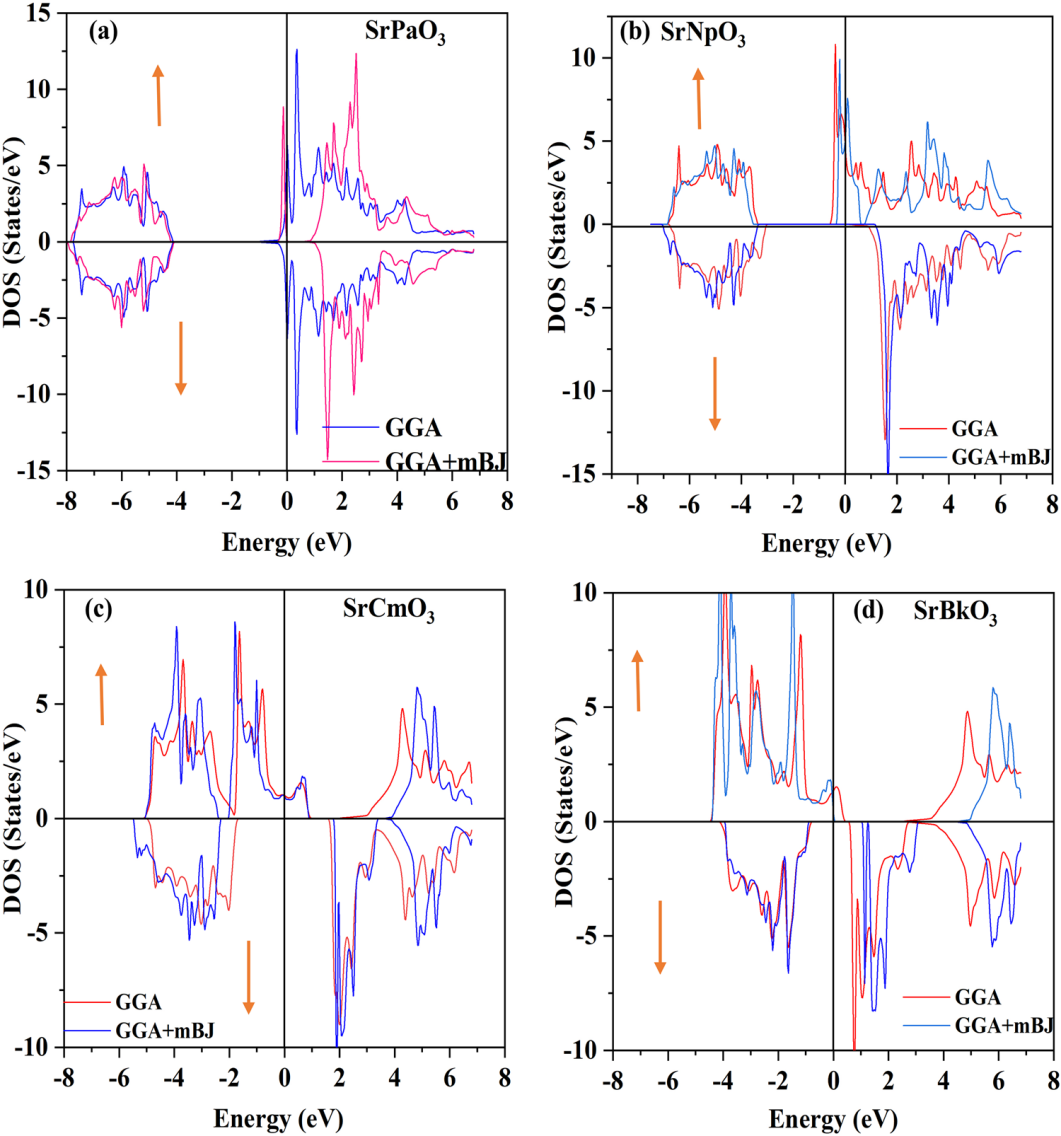
(**a-d**) Total densities of state of SrMO_3_ (M = Pa, Np, Cm, Bk) alloys.

**Figure 5:**
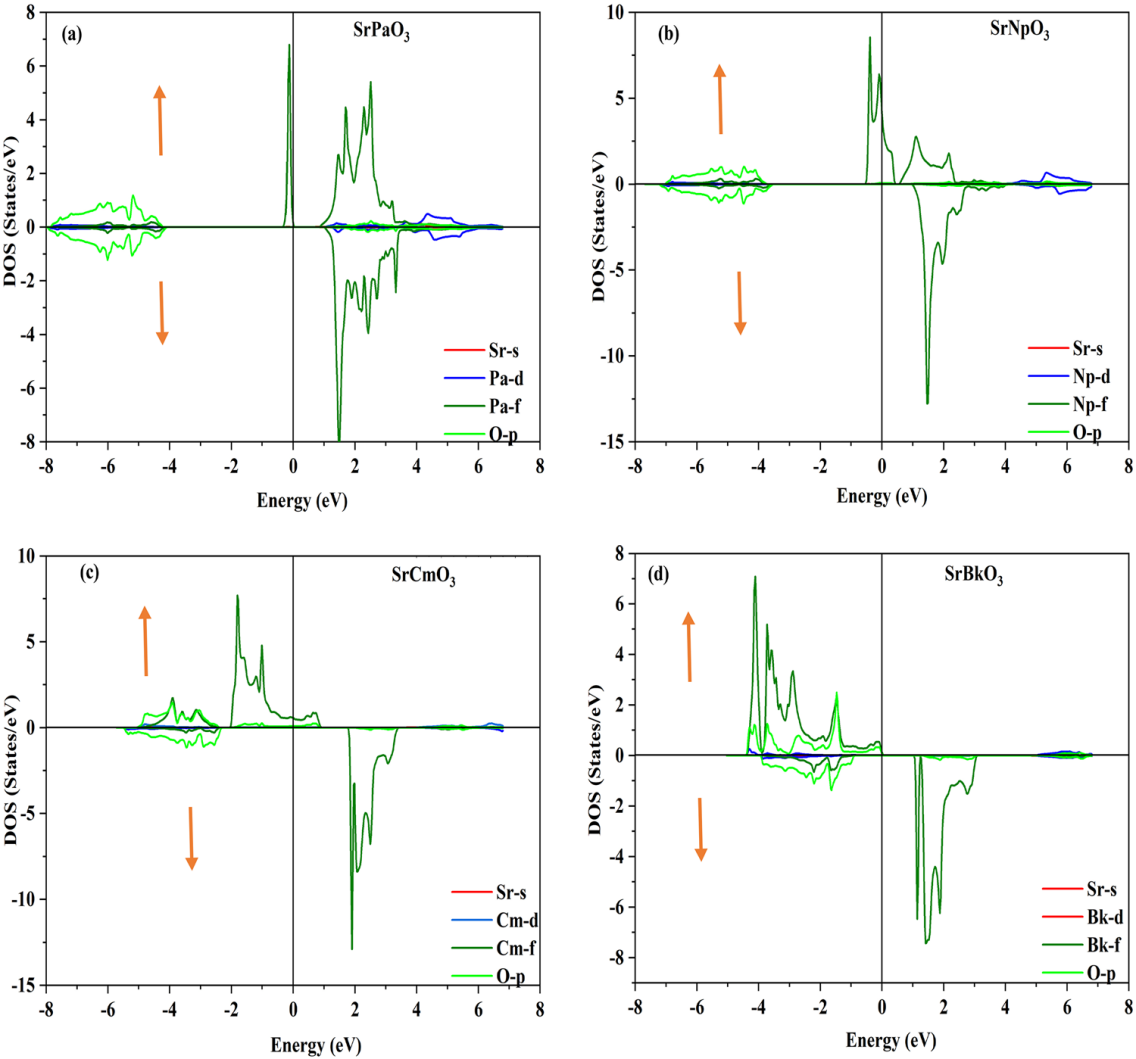
(**a-d**) partial densities of state of SrMO_3_ (M = Pa, Np, Cm, Bk) alloys.

Electronic properties of these perovskites can be evaluated from their corresponding band structure (BS) and density of states (DOS) executed in GGA and TB-mBJ functional schemes. Where, the band structure depicts allowed and forbidden energy levels of electron whereas DOS represents number of states per unit of energy^41^. To compute band profile and density of states of these materials self-consistent spin polarized calculations were performed. Primarily, within the understandings of GGA calculations the electronic structure of these alloys has been predicted which corresponds the metallic nature of SrPaO_3_ while half-metallic nature with the majority-spin corresponds to metallic and minority-spin divulges the semi-conducting nature of rest of the three materials (SrNpO_3_, SrCmO_3_ and SrBkO_3_). Therefore, the knowledge on these alloys can be further extended by evaluating their approximate band gap values. The band gap calculated within their specified electronic structures are (0.00, 3.30, 3.46 and 2.16) eV respectively for all the four alloys i.e. SrPaO_3,_ SrNpO_3,_ SrCmO_3_ and SrBkO_3_ as portrayed in Fig. 3. However, in view of the GGA potential it is not quite significant to establish a desirable band gaps of these alloys due to the involvement of localised *f-*electrons which commonly hindrances to predict the intimate electronic band structures of these alloys. Therefore, by staying within DFT, we have alternatively put forwarded the TB-mBJ potential which is considered to be better choice and pure ab initio for verifying the electronic structures of these alloys close to the experimental results. The implementation of mBJ over GGA also describes the half-metallic nature with a band gap of (4.88, 4.59, 4.17 and 2.40) eV in spin down while in contrast the spin up states unveil the metallic behaviour as the fermi level is fully occupied for SrPaO_3_ SrNpO_3,_ SrCmO_3_ and SrBkO_3_ alloys respectively with shifts in energy levels as shown in same Fig. 3. All around the band profile divulges 100% spin polarisation at the Fermi level. A little bit over view on the value of band gap for these materials is that its value is more than 2 eV so they can efficiently work at higher temperatures and voltages^42^. We have also compared our results and nature of materials with previously reported alloys as illustrated in Table 7. The interpretation of electronic properties can be further accessed through total and partial density of states (pDOS). For these materials the total DOS by GGA and mBJ approximation is plotted in Fig. 4 and from the graph it is quite evident that SrPaO_3_ reflects the same metallic and semiconducting nature by GGA and mBJ approximation respectively while remaining three alloys i.e. SrNpO_3_, SrCmO_3_ and SrBkO_3_ confirms the half-metallic nature. Further, to understand the orbital contribution of each atom we have plotted partial density of states by mBJ scheme pictured in Fig. 5, from where we can see that Pa/Np/Cm/Bk-*f* states lie at the fermi level responsible for the metallic character in up-spin while a gap in between Pa/Np/Cm/Bk-*f* and O-*p* state clearly depicts the semiconducting nature in down channel. Next, we have tried to figure out the magnetism of these alloys. The total magnetic moment is the sum of orbital and spin magnetic moments but due to the quenching of orbitals in highly correlated systems, only the spin magnetic moment is taken into consideration. The total and individual contributions of the spin magnetic moment have been enlisted in Table 6. The integral value of magnetic moment also supports the half-metallic nature of the alloys^43^. The magnetic moment arises due to the presence of unpaired electrons. In these materials the M sited atom i.e. Pa/Np/Cm/Bk has the highest number of unpaired electrons therefore, is the chief contributor towards the total magnetic moment while the least contributor towards the magnetic moment is from the first atom viz. Sr. Among the titled perovskites, SrBkO_3_ has the maximum magnetic moment of 7 µB because of the presence of maximum number of unpaired electrons. We have also compared the magnetic moment of the present materials with other perovskites as listed in Table 7. On comparison we can say that the present compounds specifically SrCmO_3_ and SrBkO_3_ exhibit higher magnetic moment than other materials thereby, pose better advances in spintronics.

**Table 6:** Magnetic moment in µB (Bohr magneton) of the materials.

Materials	SrPaO_3_		SrNpO_3_		SrCmO_3_		SrBkO_3_	
Approximation	GGA	GGA + mBJ	GGA	GGA + mBJ	GGA	GGA + mBJ	GGA	GGA + mBJ
µ interstitial	0.27	0.24	0.50	0.41	0.41	0.32	0.35	0.09
µ Sr	0.03	0.02	0.02	0.01	0.00	0.00	0.00	0.00
µ Pa/Np/Cm/Bk	0.63	0.78	2.68	2.67	5.74	5.81	6.11	6.21
µO	0.02	0.01	0.07	0.03	0.00	0.04	0.17	0.23
Total	0.88	1.00	3.00	3.00	6.00	6.00	7.00	7.00

**Table 7:** Band gap in eV, nature of electronic structure and magnetic moment in µB (Bohr magneton) of the other perovskites.

Alloys	Band gap	Nature of electronic structure	Magnetic moment
KNpO_3_	3.13	Half-metallic	2.00
KPuO_3_	3.03	Half-metallic	3.00
RaNpO_3_	3.10	Half-metallic	2.00
RaPuO_3_	3.04	Half-metallic	3.00

### Thermodynamic properties

To understand the behaviour of materials under high temperature and pressure we have calculated the various thermal properties like specific heat (C_V_), Gruneisen parameter (γ) and thermal expansion (α) within the temperature and pressure range 0–600 K;0–20 GPa by using the Quasi-harmonic Debye model^44^. Firstly, we have calculated the specific heat (C_V_) as a function of temperature at different pressure points which can be expressed as

$${C}_{V}=3nk\left[4D\left(\frac{{\theta }_{D}}{T}\right)-\frac{3{\theta }_{D/T}}{{e}^{{\theta }_{D}/T}}-1\right]$$

**Figure 6:**
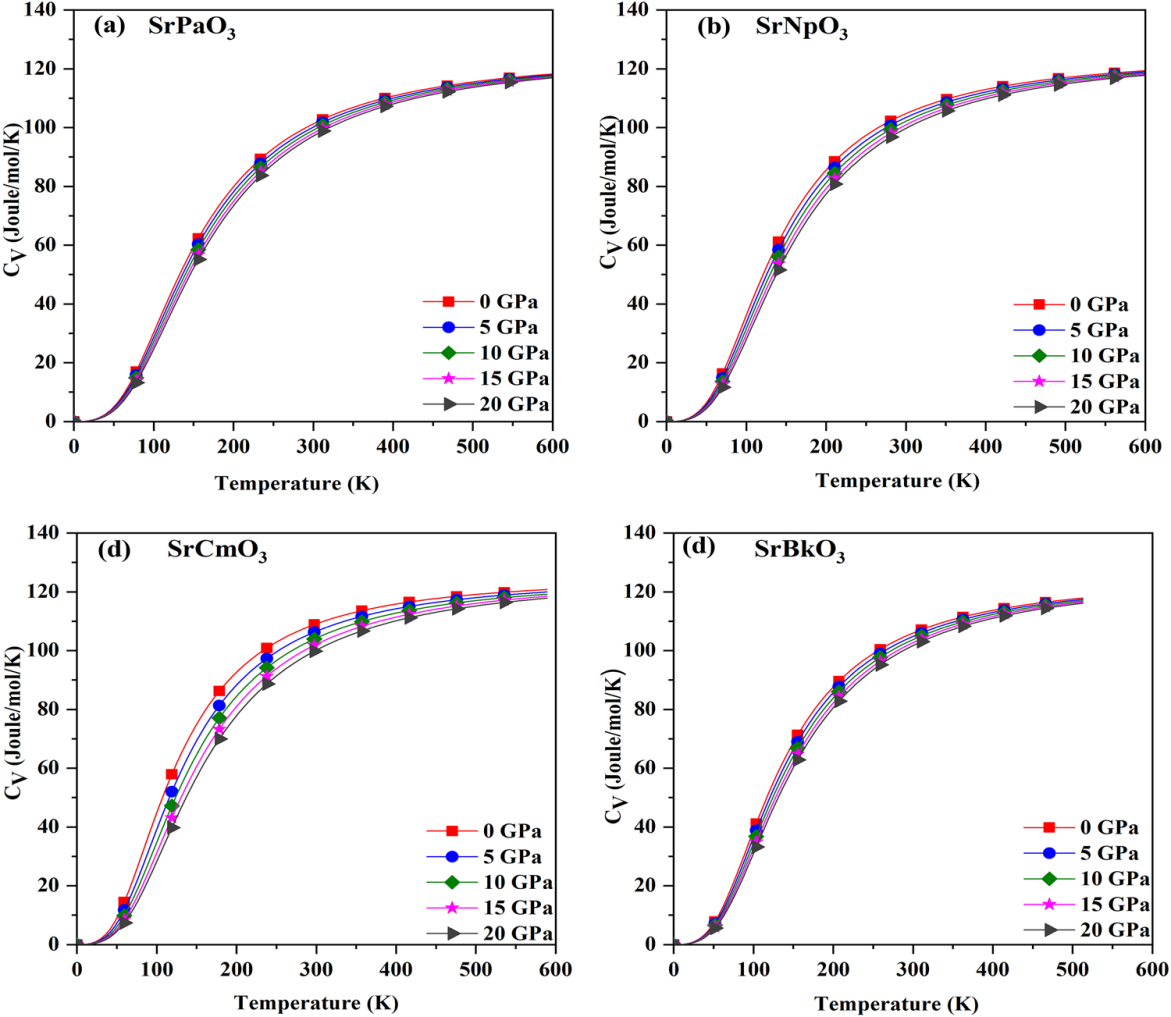
(**a-d**) The variation of specific heat (with temperature for SrMO_3_(M = Pa, Np, Cm, Bk) alloys.

The variation for these perovskites is shown in Fig. 6, and from this we can conclude that it increases with increase in temperature and then gain constant value at higher temperature obeying the Dulong-Petit law^45^. The increasing trend at lower temperatures is due to the increase in atomic vibrations with increase in temperature. Whereas, at higher temperatures, the molecules have more thermal energy and are able to move freely. So, increase in the average thermal energy becomes less effective. The value of C_V_ for these materials at room temperature are enlisted in Table 8 and from that we can infer that SrCmO_3_ has maximum value of C_V._

**Table 8:** The calculated value of specific heat (C_V_ in J/mol/K), Gruneisen parameter (γ), and Thermal expansion (α) in 10^–5^/K at room temperature**.**

Alloy	C_*V*_	γ	α
SrPaO_3_	101.82	1.54	1.05
SrNpO_3_	104.98	1.84	1.57
SrCmO_3_	109.46	3.46	4.02
SrBkO_3_	106.03	1.58	1.42

**Figure 7:**
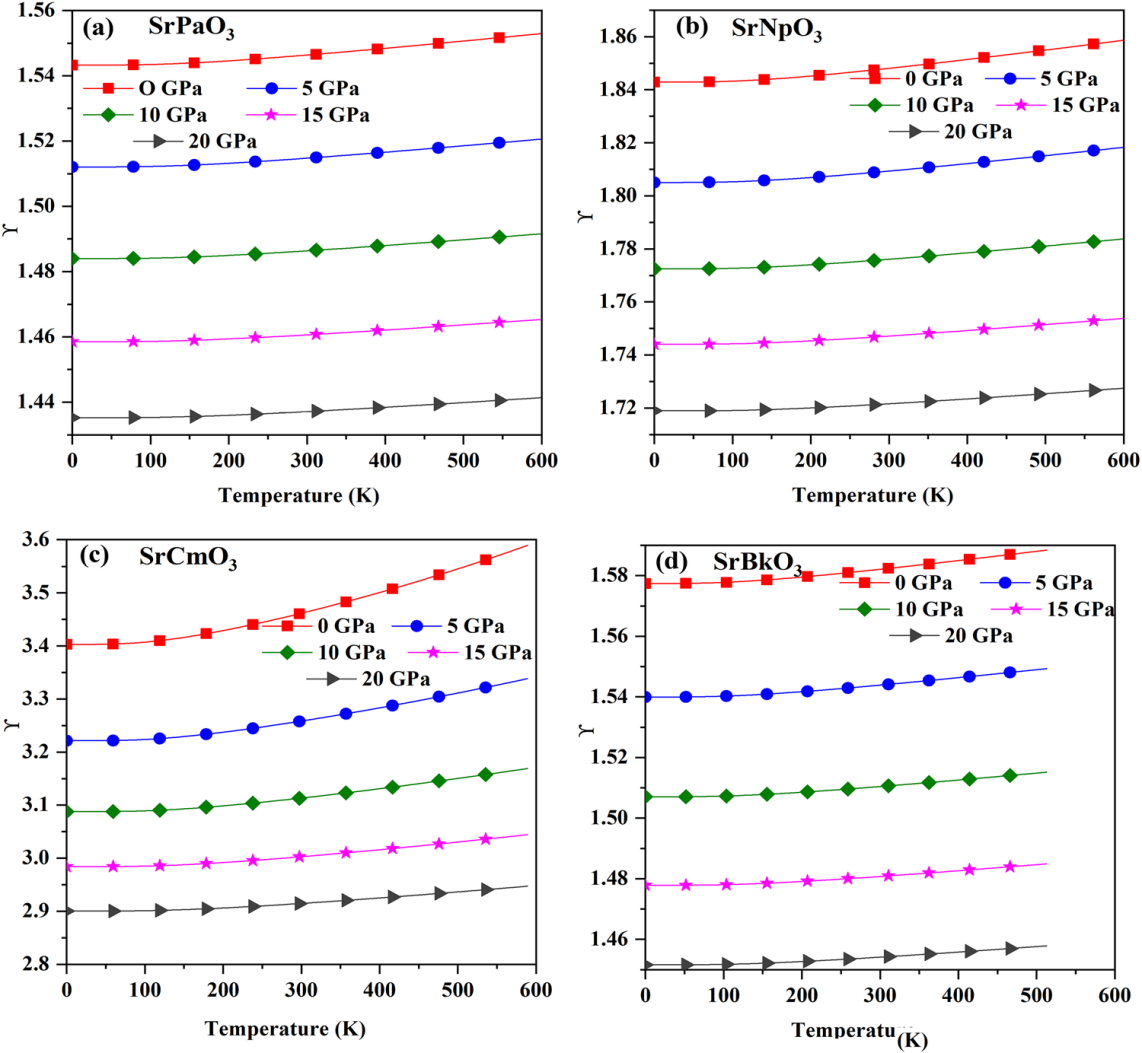
(**a-d**) The variation of Gruneisen parameter (γ) with temperature for SrMO_3_(M = Pa, Np, Cm, Bk) alloys.

Next, we have calculated the Gruneisen parameter (γ) which is a dimensionless quantity. It tells about the thermal state of material. Also, it gives information about the variation of anharmonicity in the crystal lattice^46^. Here, for these materials we get a trivial increasing trend with increase in temperature but we have observed that there is a significant change with change in pressure as depicted in Fig. 7, so this implies that pressure effect outperforms the temperature effect. The calculated value of Gruneisen parameter (γ) are enlisted in Table 8.

**Figure 8:**
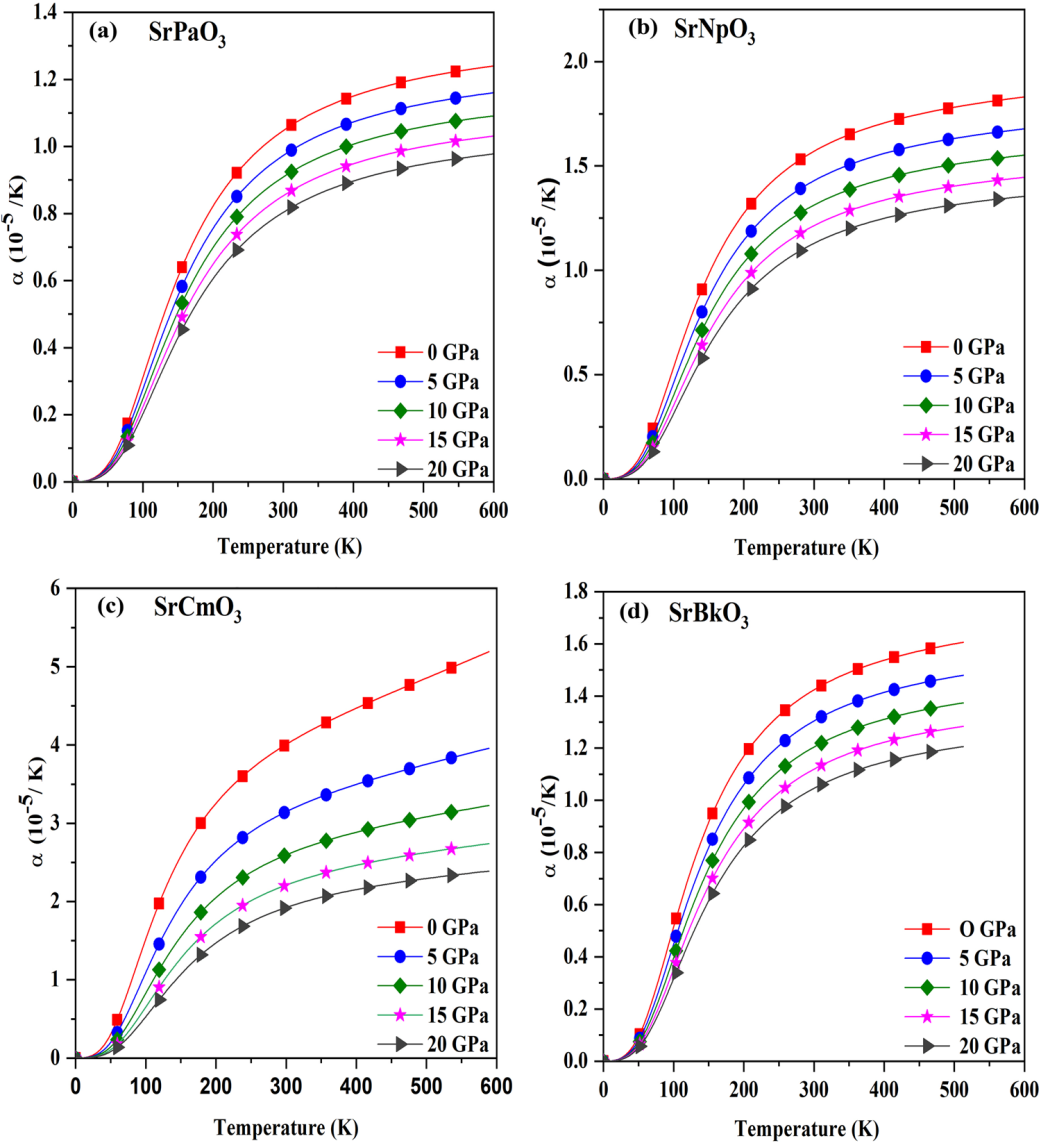
(**a-d**) The variation of thermal expansion (α) with temperature for SrMO_3_(M = Pa, Np, Cm, Bk) alloys.

After that, we have calculated the thermal expansion coefficient (α) which can be expressed as $\alpha =\gamma {C}_{V}/{B}_{T}V$, to determine up to what extent expansion can take place. Figure 8, shows the variation of α at different pressures and temperature. From this figure it is clear that it increases with rise in temperature because with surge in temperature, the bond strength decreases resulting an increase in thermal expansion while with increase in pressure it decreases, because pressure enhances the bonding among the atoms and thus atoms are held tightly so thermal expansion decreases with an increase in pressure. The value of α at room temperature has been enlisted in Table 8.

### Thermoelectric properties

As one-third part of consumed energy in everyday use is lost to space in form of waste heat, because of the limited efficiency of electronic devices. Hence, there is a need to design smart devices that can harvest at least some part of the wasted heat productively. Here comes the role of thermoelectric devices which can convert waste heat into usable energy. To understand the thermoelectric behavior, we have evaluated the different transport parameters like Seebeck coefficient (S), electrical conductivity (σ/τ), thermal conductivity (κ) and power factor (PF) as a function of temperature by using the semi- classical Boltzmann transport theory^47^.

**Figure 9:**
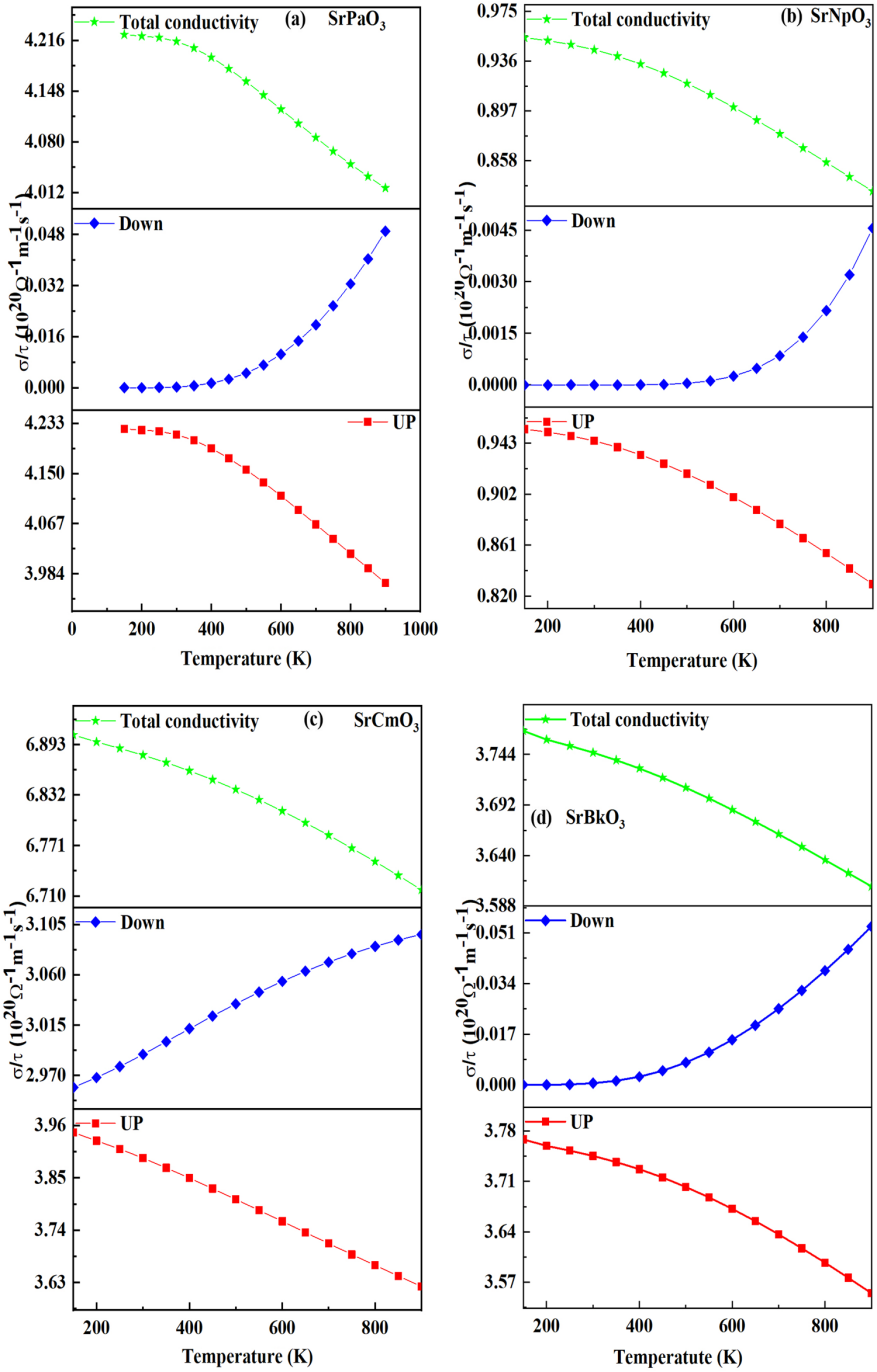
(**a-d**) Variation of electrical conductivity for both the spins and total conductivity against temperature for SrMO_3_(M = Pa, Np, Cm, Bk) alloy**s.**

**Figure 10:**
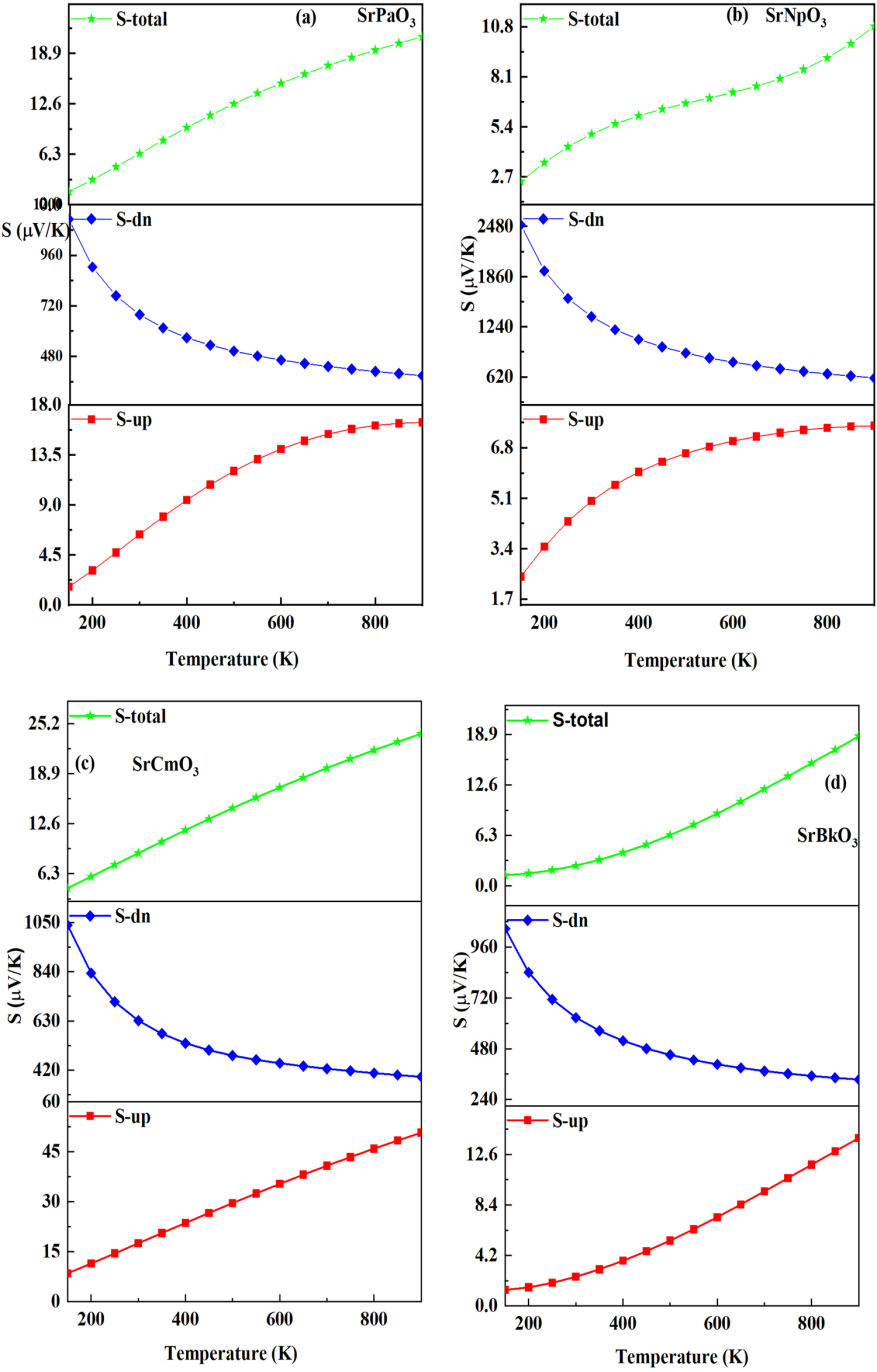
(**a-d**) Variation of seebeck for both the spins and total seebeck against temperature for SrMO_3_(M = Pa, Np, Cm, Bk) alloys.

At first, we have calculated electrical conductivity reported in Fig. 10, for up and down spins and we observed that electrical conductivity for all the materials in up spin decreases with increase in temperature because when temperature increases, the vibration of metal ion increases so results in increase in resistance of metal and hence, decrease in conductivity so, assuring the metallic nature of the materials while in opposite channel the conductivity increase with increase in temperature so reflecting the semiconducting nature of the materials. The reason is attributed due to the fact that with increase in temperature, number of electrons from the valence band jump to conduction band resulting in increase in conductivity of the material. The calculated values of electrical conductivity at room temperature and at maximum temperature for both the spins are listed in Table 9. Here, we have also calculated the total electrical conductivity of these half-metallic compounds which is the sum of electrical conductivities of both spin channels. The variation is shown in same Fig. 9.

**Table 9:** The calculated value of electrical conductivity in 10^20^ Ω^-1^ m^−1^ s^−1^ for both the spins.

Alloy	Spin state	(σ/τ) (300 K)	(σ/τ) (900 K)
SrPaO_3_	Up	4.21	3.96
	Down	0.0002	0.04
SrNpO_3_	Up	0.94	0.82
	Down	0.0001	0.004
SrCmO_3_	Up	3.89	3.62
	Down	3.00	3.09
SrBkO_3_	Up	3.74	3.55
	Down	0.0005	0.05

**Figure 11:**
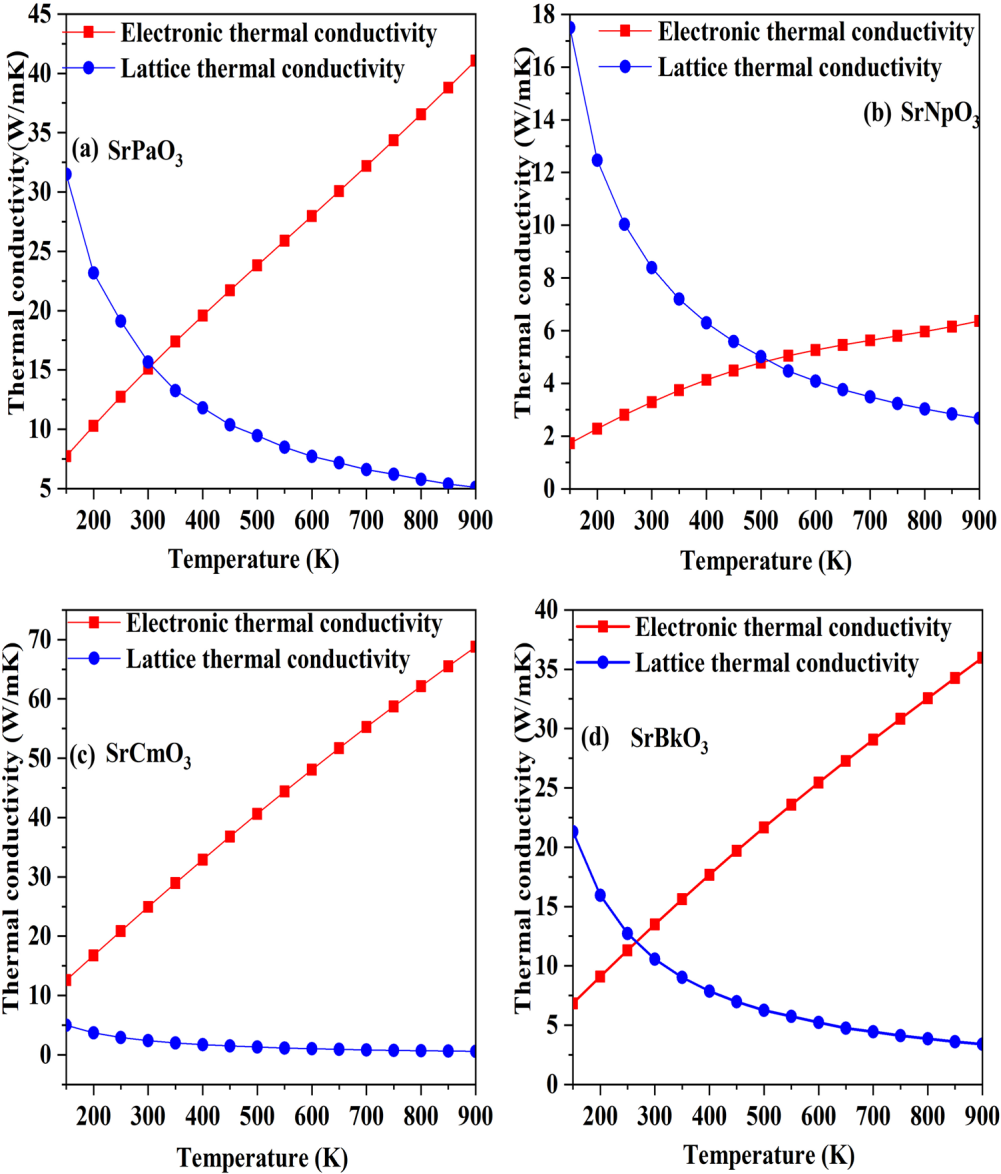
(**a-d**) Variation of thermal conductivity (electronic and lattice thermal conductivity) against the temperature of SrMO_3_(M = Pa, Np, Cm, Bk) alloys**.**

After that, we have calculated the Seebeck coefficient for both the spins pictured in the Fig. 11. Numerically it is defined as, $\Delta S=\frac{\Delta V}{\Delta T}$ where ∆T is the temperature gradient. From the up-spin graph, we observed the increasing trend for these materials because for the case of metals Seebeck increases with increase in temperature. However, in down channel the value of Seebeck decreases as temperature rises due to the creation of more charge carriers which scatter each other effectively thus causes the Seebeck to decrease. The decreasing nature descripts the semiconducting nature of these materials. Subsequently, we have computed the total S via two current models^48^. The total S through this model is given by the following equation $S = \frac{{[S^{ \uparrow } \sigma^{ \uparrow } + S^{ \downarrow } \sigma^{ \downarrow } ]}}{{[\sigma^{ \uparrow } + \sigma^{ \downarrow } ]}}$. The variation of total Seebeck coefficient with temperature is plotted in Fig. 10. From the graphs it is clear that total S increases with an increase in temperature for these materials. The values of Seebeck coefficient are positive in whole range indicating the p type nature of these materials. The calculated values of Seebeck coefficient for both the spins at room temperature and at 900 K are listed in Table 10.

**Table 10:** The calculated value of the Seebeck coefficient (in microvolt/K) in both the spins for SrMO_3_ alloys.

Alloy	S (300 K)		S (900 K)	
	Spin up	Spin down	Spin up	Spin down
SrPaO_3_	6.34	678	16.42	388
SrNpO_3_	5.00	1365	7.50	608
SrCmO_3_	17.50	631	50.63	391
SrBkO_3_	2.42	627	13.90	335

Next, we have calculated another important parameter which is thermal conductivity, which mainly arises because of two processes i.e. drift in the electrons and holes (${\kappa }_{e})$ and secondly due to travelling phonons (${\kappa }_{l}$). The variation with temperature for these two parts is shown in Fig. 11, and from the graph we can infer that the electronic thermal conductivity follows an increasing trend because with increase in temperature the molecular vibrations increases. It is a drawback of Boltz Trap that it cannot calculate the lattice part of thermal conductivity so to calculate the lattice thermal conductivity, we have used Slack’s equation enumerated as: $\kappa_{l} = \frac{{A\theta_{D}^{3} V^{1/3} m}}{{\gamma^{2} N^{2/3} T}}$^49^. From the equation it is clear that the Debye temperature (${\theta }_{D}$), Gruneisen parameter (γ), temperature (T), volume (V), average molar mass per atom (m), and the number of atoms per unit cell (N) effect the lattice thermal conductivity so by the means of these factors we can evaluate the lattice thermal conductivity. From the graph we can see that it decreases with increase in temperature. It is due to the increase in phonon scattering, where phonons interact with each other and transfer energy by collisions. So, there is a reduction in mean free path of phonons, hence, heat transportation reduces, resulting in decrease in thermal conductivity. So, we can conclude that these materials are apt for utilising the waste heat.

**Figure 12:**
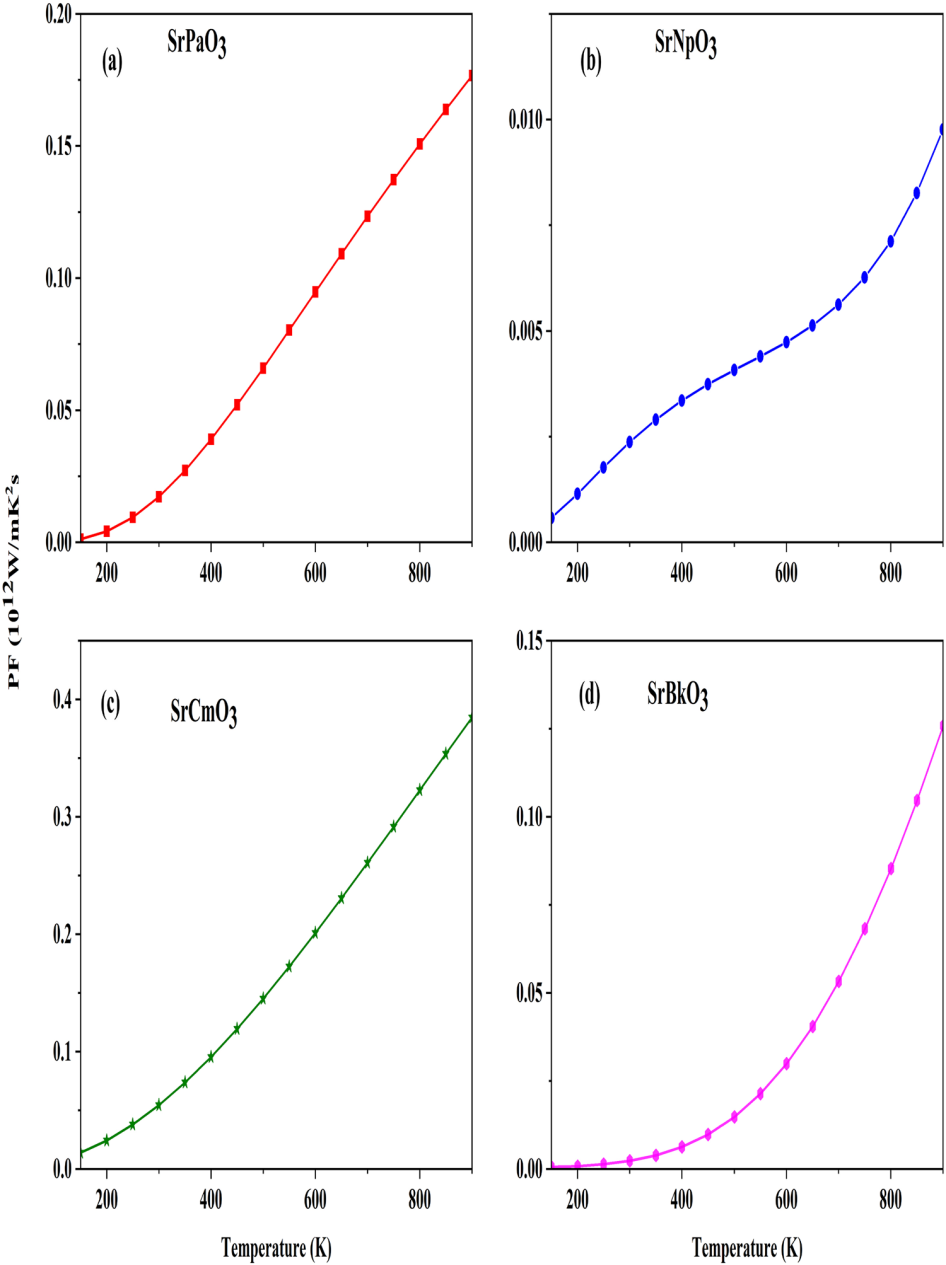
(**a-d**) Variation of power factor against the temperature of SrMO_3_(M = Pa, Np, Cm, Bk) alloys**.**

At last, we have also evaluated the power factor which dictates the thermopower generation which we have computed with the help of total Seebeck coefficient and total electrical conductivity. The variation with temperature is portrayed in the Fig. 12, which shows increasing trend with rise in temperature. The increasing trend of these parameters designates that these materials are suited for thermoelectric device applications. The study indicates that the present materials have high thermopower along with half-metallicity so they could potentially be used for spintronics and thermoelectric applications.

## Conclusions

The DFT prediction of SrMO_3_ (M = Pa, Np, Cm, Bk) related to their structure, electronic, magnetic, mechanical, thermal and transport properties have been summed up. The tolerance factor, structural optimizations and cohesive energy calculations defines the stability of the alloys. The mechanical parameters divulge the ductile nature is preserved within their lattice structures. The band structures together with their corresponding density of states explains the half-metallic nature of the alloys with a band gap of (4.88, 4.59, 4.17 and 2.40) eV in spin down while metallicity is retained in up spin. These alloys could be potential candidates for MRAM devices because of their high magnetic moments and half‐metallic character. Also, the effect of temperature and pressure on different thermal parameters propose their applicability at higher temperatures. And at last, thermoelectric parameters like electrical conductivity, Seebeck coefficient, thermal conductivity and power factor have been investigated in the temperature range of 150–900 K which showcase a decent value of these parameters. Hence in nutshell, these materials projects a potential stand in spintronics and solid-state device applications.

## Data Availability

The data would be available from the corresponding author on a reasonable request.
